# There's Safety in Numbers

**Published:** 1971-01

**Authors:** Alan B. Raper

**Affiliations:** Consultant Pathologist, United Bristol Hospitals


### There's Safety in Numbers

**Alan B. Raper, M.D., F.R.C.P., F.R.C.Path.**

Consultant Pathologist, United Bristol Hospitals

The Bishop had delivered his reassuring opinion—"There's safety in Numbers"; but the young curate beset by a clutch of spinsters respectfully submitted that for his part he saw much more in Exodus. We must leave him now, but return to him later to see how his problem and its solution compare with those of the modern physician.

All natural phenomena are no doubt quantifiable, even if for some, such as the emotions and especially value judgements, we have not yet discovered the notation. Fortunately I do not have to defend or attack that hypothesis, because the measurements that are actually used in medicine are the only ones that I wish to consider. And the fact that I work in a laboratory is at once the reason for my interest in measurable phenomena and the basis for any prejudices or critical views that I may entertain.

Anyone who issues a numerical report from a laboratory, and anyone who receives one, is entitled to ask several questions. First, how accurate are these figures? Second what do they tell me that is relevant to the enquiry that elicited them? Was it worth getting to know (or determining) these figures? May not the bare figures, however accurate, in fact mislead? And there is a final question, following from an answer to the others; could I have obtained (or given) the information needed without resort to quantitation?

How safe, indeed, are our numbers, and what use do we or could we legitimately make of them? At one extreme there are those, both clinicians and laboratory workers, who hardly ever ask this question, and accept laboratory figures as the simple truth. They are undoubtedly mistaken. At the other extreme there are those who habitually disbelieve or disregard laboratory measurements in favour of clinical judgement, but ask for or permit the measurements to be made. They are clearly infirm of purpose. Most of us take an intermediate position, and are interested to know how far we can rely upon the figures supplied. So where does the truth lie? In fact the answer is different from every metric test, although several broad groups can be recognised.

Chemical determinations are the result of measurements made at the end-point of, or at several intervals during, a reaction. Their credibility depends solely on meticulous care in the performance of the test; there is no other check. Titrations (as in immunology) that depend on step-wise dilutions have a precision limited by the size of the "steps". Counts of discreet objects such as leucocytes depend for their accuracy on the making of dilutions and the mathematics of the enumeration. If we limit our consideration to the tests only, setting aside all other sources of error, every test of these sorts has its own probability of error. Sometimes this probability is well known to the pathologist. If it is, a logical method of reporting would state the figure followed by "$\pm x$" where $x=2$ standard deviations, so that "$\pm x$" defines the limits outside of which the true results would be likely to lie only once out of a total of 20 trials. In some situations it might be as well not to quote the figure at all, but to indicate how far in terms of standard deviations, and in what direction, the result differs from the normal mean. For many determinations, too, it would be sensible to round off the result to the nearest multiple of a power of 10, depending on the order of magnitude selected for the figures of the report. However, these things are rarely done, chiefly because they introduce a further step or calculation that is different for each sort of test; and the lack of this practice can be counted as a criticism of pathologists, and as a reply to many of their criticisms of clinicians who may misinterpret results. But even if they were done it would still not reveal two very important sources of error which can widely overshadow the inherent errors of the test itself.

The first serious source of error lies in the many circumstances surrounding the test. At every stage there is danger; the collection of and the right treatment of specimens, alterations due to delay in forwarding or processing the specimens, errors in transcription of the patient's details in ward or laboratory, or of the figures in making up the final report. No "quality control" of the tests can detect these. They may not be frequent, but every observant laboratory worker knows that they occur almost daily, and that the greater the volume of work being handled, the greater the **proportion** of these errors. Not only that, but the effective watchfulness of the pathologist decreases with the volume of work. One has only to work in units of different sizes to appreciate the importance of scale; many of the advantages of handling tests in large numbers are more than counterbalanced by the lack of critical personal attention. Of course it is true that an increased volume of work can be achieved by the use of mechanized equipment, but it is important to realize that while mechanization can free laboratory workers from strain, and so increase their effective vigilance, this only applies if the volume of work remains much the same. In practice, mechanization allows, indeed invites, more tests, and soon the original prevalence of this sort of error is restored.

The second source of error lies within the test; it is that element of human fallibility that is not allowed for in quoting the standard deviation of an estimation. Misunderstanding of methods, lapses of attention, the acceptance of wrong reagents or unsuitable specimens, and sheer ignorance, are some factors. Who, outside the laboratory, knows whether the figures he reads were culled by a girl who has just left school, or by an experienced worker devoted to this particular examination? These errors are very difficult to detect and to exclude. Clinicians detect them when the result is incompatible with all the rest that they know about the patient. Pathologists are continually detecting them on the same principles, in their own work as well as in that of their technicians, and capable technicians are constantly on the watch for them; again, the process is an exercise of judgement applied to all the available information. And once again the use of mechanized equipment reduces these errors. Also, that aspect of quality control that surveys the day's results detects any systematic error in a test (for example, the use of an incorrectly made-up reagent); but it does not identify single errors. "Rogue" results can occur even with electronic equipment. I have known a surge of electrical energy to cause an electronic counter to register five times the true leucocyte count. So even if we are satisfied with the daily distribution curve for a series of tests, and if we have included a control at every 20th run, and have calculated the **chances** of error at a very low figure, we still cannot tell whether Test No. 53 (which happens to be on your blood, or your child's) differs slightly or widely from the true value. We cannot tell, that is, unless we have some collateral evidence applicable to that particular sample. The danger is greatest in chemical tests considered in isolation; there is no way for instance of checking whether a given blood sugar is correct except by repeating the test, by another method if necessary. Fortunately, however, when a group of chemical estimations is made on one specimen, an abnormal result in one of them might stand out as incompatible with the rest, so that there is some scope for the pathologist's judgement. There is less danger in haematological tests, for here the collateral evidence can be supplied by a totally independent method, the visual inspection of a blood film; this is true quality control and has nothing to do with means and standard deviations. As an aside, I put it forward as a delightful example of doublethink that we should use the expression "quality control" when we really mean "quantity control".

But now, suppose all our figures to be credible, there are still some questions to be answered. Was it worth obtaining the figures? Do they contribute anything of value? Often, of course, we cannot answer that until we see the figures, and thus if there is no other route to the information we want, to obtain the figures is justified. It is in fact a matter of judgement whether to obtain the figures, and whether to trust them alone to solve one's problem. Leucocyte counts provide many examples of this sort of situation. For instance, no experienced surgeon would await a leucocyte count to help him to decide the diagnosis of an acute abdomen, or be diverted in his judgement by a normal figure which might well conceal the fact that gross changes were present in the morphology of the cells. And it is by no means a paradox that the lure of a leucocyte count is more dangerous to a less experienced surgeon, who would do better to call in superior clinical judgement rather than laboratory enumeration. In any case, the eye of the pathologist can supply much more information than a count—as well as estimating the count with a degree of accuracy quite sufficient for most clinical situations of this sort. Moreover the considered statement of a pathologist, who knows only too well the possibilities for error in his laboratory, is a value judgement that far outweighs in credibility any figures that may have been produced. The two things differ in much the same way as a hand-signal from a driver differs from the message of his mechanical traffic indicator; only the former is really credible, because it is the visible expression of a **mind** at work, while to trust to the latter may be to invite disaster.

At this point it is worth reflecting upon the reasons for the multiplicity of unnecessary tests. There are four main considerations. Some tests are ordered through inertia; in the 1920's it was new, exciting, and possible to have a red cell count, but now it is rarely needed. This, like several other numerical tests, has served its purpose; by its help the field has been cleared, and judgement by other means has been informed, so that it can now operate without recourse to the figures. Secondly, some tests are now ordered because they are so easily provided by machines. There may be intellectual objections to this, but there are few practical ones. However, it should not escape notice that machines are in general produced and promoted not always to fulfil a need, but simply because someone with a mechanical bent has devised a certain means of using a machine, which can now earn cash for the promoter and confer prestige on the owner irrespective of its true value. Then come the "steam-hammer for a nut" tests. The worst examples are tests ordered when the required answer could easily be obtained (say on the ward) by simply looking at a specimen; visible melaena or a visibly pale stool do not generally require laboratory tests; threadworms or the segments of tape worms can be seen by all but those too lazy to look. These are not of course numerical tests. A reticulocyte count is, and I have known it to be asked for in order to answer the question "Is the patient bleeding?"; the effort is wasted, for whatever the result it can provide neither a Yes nor a No to the question. Lastly come the many understandable causes of unnecessary tests, such as "for completeness", "as a base line", "because this is a teaching hospital", "because I may be blamed if I don't do it", "it might turn something up" (on the bran-tub principle), "I have read that the X test is positive in Y disease; I know this patient has Y disease, therefore I shall do an X test". One can sympathise in various degrees with each of these reasons, though none of them is a real justification. It is also possible to point to unexpected diagnoses arrived at through apparently useless tests, although against this rare bonus is to be set the daily distraction from useful work by profitless activity.

There is an increasing demand for laboratory "profiles", that is, a battery of tests selected for their general usefulness and their suitability for automated analysis, regardless of the patient's symptomatology. The intention is that abnormalities, perhaps unsuspected, should be detected early, or at any rate coincidentally with the performance of other more specific tests. Little time or effort is wasted in producing the full "profile". Abnormal findings are of course rare, but could be useful; they thus fall under the heading of the rare bonuses mentioned above. Is it worth obtaining the profile? There is one strong argument in favour of this. It is that the normal range for very many tests is not known with certainty. Many "normal ranges" have been obtained by examining relatively few subjects, often young persons such as soldiers, medical students, or technicians. Analysis of large numbers of profiles could re-define the normal ranges (e.g. for the two sexes, and for various age groups). When this has been done, abnormal results will be more clearly identified, and perhaps then linked to clinical observations not regarded as critically significant hitherto. My own forecast is that while this goes on over the coming years, the "bonuses" will be eagerly accepted, and thereafter the "profile" will fall into disuse (except among the unthinkingly conservative), in much the same way as has the mindless request for "full blood count".

To describe the many irrelevancies and the many possibilities for error in our figures is admittedly to paint too gloomy a picture. Of course the vast majority of tests are sufficiently accurate, and a great many of them are useful; also most of the minor errors (usually undetected) do not matter. But the pathologist is still worried by the thought that the inexperienced may act, and act wrongly, even upon the accurate figures that he supplies, when if his own or another experienced judgement had been called for, the interpretation might have been different, or supplied without the need for quantitation. Further, to turn out accurate figures is not necessarily an unadulterated good, if by clogging up the day's work-load this means that attention cannot fully be given to tests that really matter.

It will now be apparent that I am advocating the use of numerical tests only when more reliable (because more all-embracing) judgement cannot be deployed instead of them, and only when they can be expected to give information that will really contribute to still sounder judgement. At this point, I hope you have not forgotten the curate. In his special circumstances he may well have been right. But if his problem had been that of a clinician proposing to seek help from a medical laboratory, he should surely have sought safety not in Numbers or Exodus, but in Judges.

